# Supplementary material for: Quantum Disordered State of Magnetic Charges in Nanoengineered Honeycomb Lattice
Source: Adv Sci (Weinh). 2021 Feb 5;8(6):2004103. doi: 10.1002/advs.202004103 (PMC7967061; doi:10.1002/advs.202004103)
Supplement: Supplementary file 1 — Supporting Information [file ADVS-8-2004103-s001.pdf]

© 2020 Wiley-VCH GmbH

ADVANCED SCIENCE
Open Access

Supporting Information

for *Adv. Sci.,* DOI: 10.1002/advs.202004103

Quantum disordered state of magnetic charges in nanoengineered honeycomb lattice

*G. Yumnam, Y. Chen, J. Guo1, J. Keum, V. Lauter, D. K. Singh**

Supporting Information: Quantum disordered state of magnetic charges in nanoengineered honeycomb lattice

G. Yumnam[1], Y. Chen[1], J. Guo[1], J. Keum[2], V. Lauter[2], D. K. Singh[1*]

[1]*Department of Physics and Astronomy, University of Missouri, Columbia, MO 65211*

[2]*Oak Ridge National Laboratory, Oak Ridge, TN 37831*

1 Next-nearest neighbor exchange energy vs dipolar interaction energy

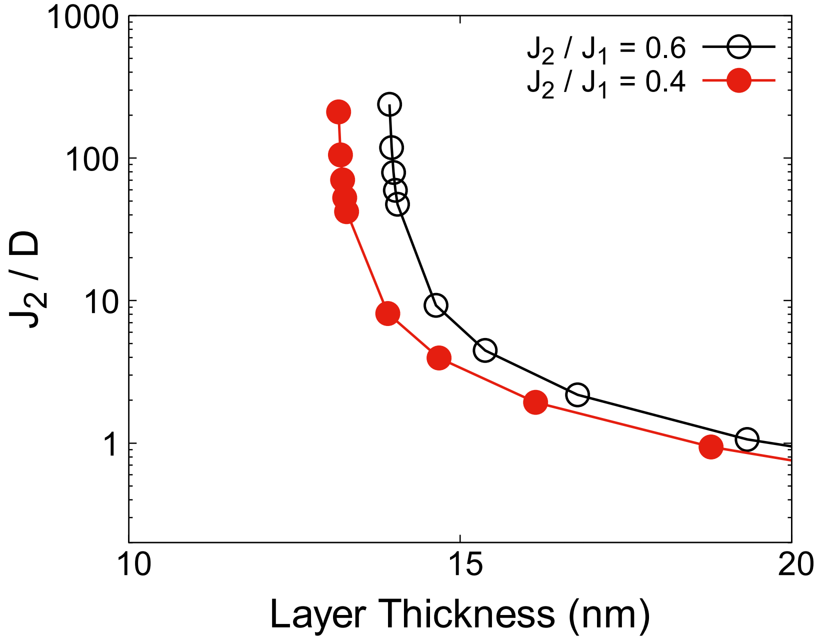

Figure **S1**: The ratio of J_2/D as a function of permalloy layer thickness for various J_2/J_1.

We have followed magnetic charge Hamiltonian as described in reference [14], which in-

cludes all three terms, eg. nearest neighbor (J_1) and next-nearest neighbor (J_2) exchange interaction as well as magnetic dipolar (D) interaction. As we can see in Figure **S1**, J_2 is much larger than D in our honeycomb lattice (element length $\sim$ 12 nm) of moderate thickness, $\sim$ about 10 nm. Thus, J_2 term is more important than dipolar interaction in our honeycomb lattice. Our calculation is most likely not valid in large element size (about a micrometer element size) honeycomb where dipolar interaction is much stronger. In our case, the dipolar interaction is $\sim$12 K.

2 Exchange energy of nearest and next-nearest neighbor type at different layer thickness

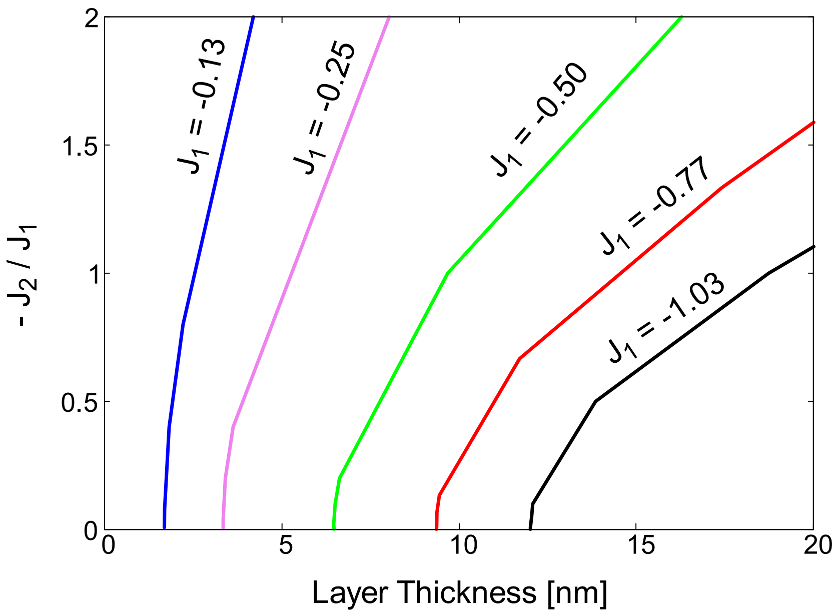

Figure **S2**: The ratio of J_2/J_1 as a function of permalloy layer thickness for various J_1.

The nearest neighbor and the next-nearest neighbor exchange interactions were estimated as a function of permalloy layer thickness by considering the magnetostatic energy of magnetic charges located at the vertices of honeycomb lattice. The system's energy for a well-converged

30×30 kagome superlattice was calculated using Monte-Carlo method under the Heisenberg spin-model. Here, the nearest-neighbor exchange interaction as antiferromagnetic ($J_1 < 0$) and the next-nearest neighbor exchange interaction is ferromagnetic ($J_2 > 0$). Fig. **S2** shows the plot of J_2/J_1 as a function of permalloy thickness for different values of J_1. The length of honeycomb element is 12 nm.

3 Magnetization measurements

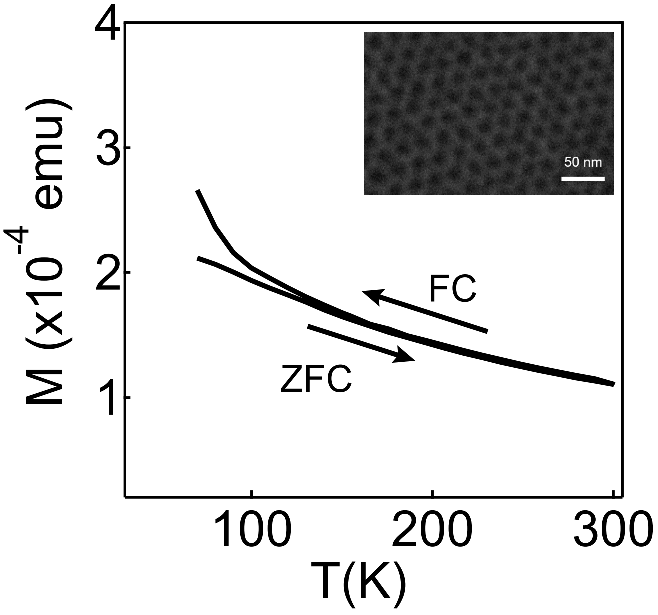

Figure **S3**: The plot of magnetization with respect to temperature, obtained using ZFC/FC protocol. Inset: Scanning electron micrograph of the magnetic honeycomb lattice.

We have performed magnetization measurements of our honeycomb lattice using Quantum Design SQUID magnetometer. Magnetic measurement was performed in 50 Oe magnetic field, applied in plane to the sample. As we can see in Fig. **S3**, the system exhibits paramagnetic behavior

throughout the measurement range. This is consistent with estimated magnetization from PNR measurements, as shown in Fig. 2e, where magnetization nearly doubles between 200K and 30K. But no signature of long range order or blocking characteristics was detected. It further suggests that the system is behaving as a cooperative paramagnet even at low temperature.

4 X-ray reflectivity

The X-ray reflectivity (XRR) of the Permalloy honeycomb lattice was measured using Cu-Kα_1 with wavelength of 1.5406 Å. The XRR is well-fitted with an X-ray scattering length density (SLD) profile as shown in Fig. **S4**. The depth-profile estimated from XRR is consistent with neutron reflectivity fitting results.

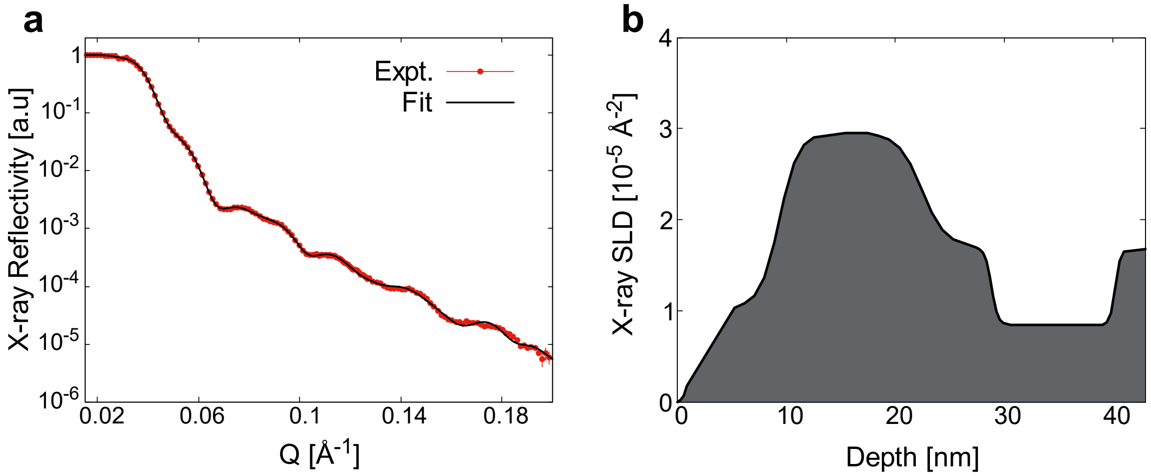

Figure **S4**: **(a)** The X-ray reflectivity and **(b)** X-ray scattering-length density profile.

5 Specular Reflectivity fitting at different temperatures

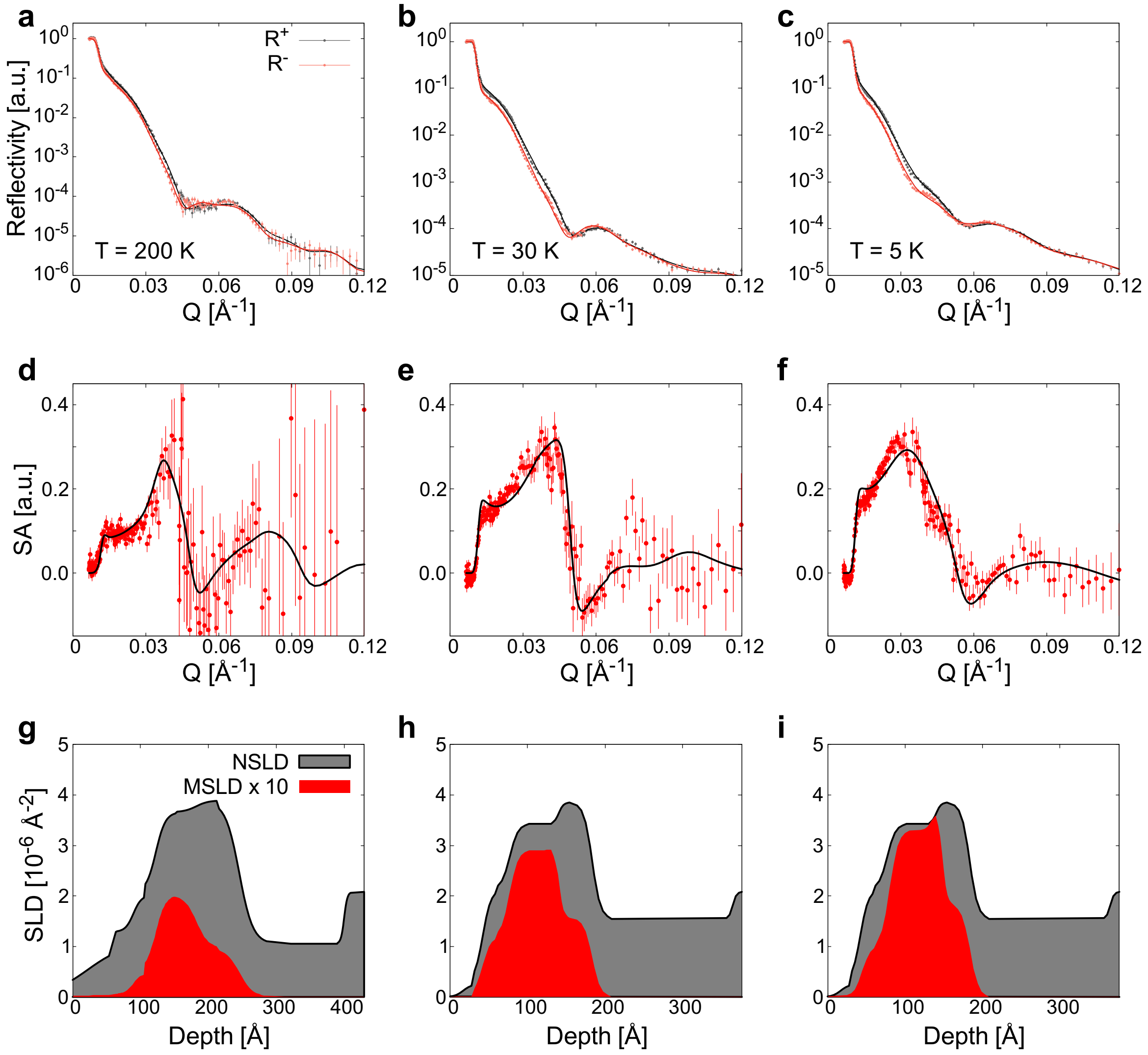

Figure **S5**: **(a-c)** The measured and fitted specular reflectivity for neutrons with spin-up (R^+) and spin-down (R^-) polarization. **(d-f)** Spin-asymmetry (SA = $(R^+ - R^-)/(R^+ + R^-)$) as function of wave-vector transfer (Q). **(g-i)** Nuclear (NSLD) and magnetic (MSLD) scattering length density as a function of depths at different temperatures.

The spin polarized neutron reflectometry measurements were performed at BL-4A at SNS,

ORNL at various temperatures. Plot of specular reflectivity is well fitted for structural parameters obtained from XRR. The scattering length density (SLD) profile used in our reflectivity fitting incorporates both nuclear (NSLD) and magnetic (MSLD) contributions.

6 Degenerate spin configurations at low temperature

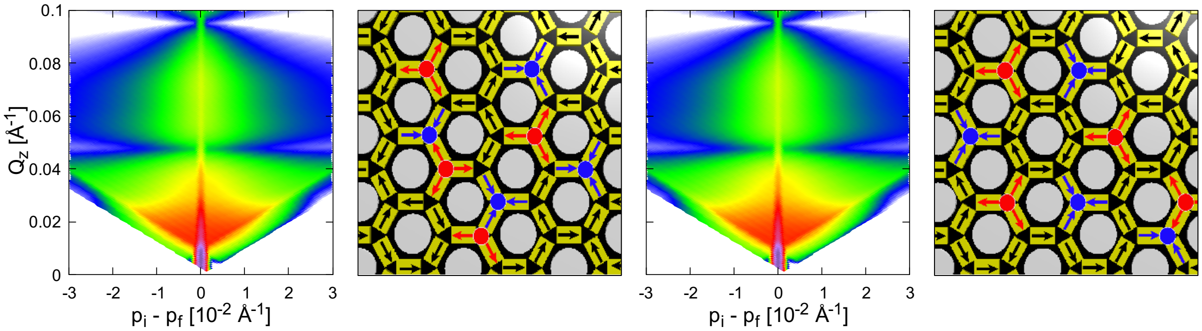

Figure **S6**: Numerically simulated off-specular PNR profiles for degenerate magnetic charge configurations.

The off-specular reflectivity at low temperature was simulated within the Distorted-Wave Born Approximation (DWBA). For the off-specular profile. We observe that the off-specular reflectivity profile is indistinguishable for two degenerate spin configurations, with an equal number of $\pm$3Q and $\pm$Q magnetic charges distributed across the lattice.

For completeness, we have also simulated the off-specular reflectivity for pure states of spin ice and spin solid configurations. As shown in Fig. **S7**, the off-specular patterns due to pure states are very different from the mixed charge configurations, as shown in Fig. **S6**.

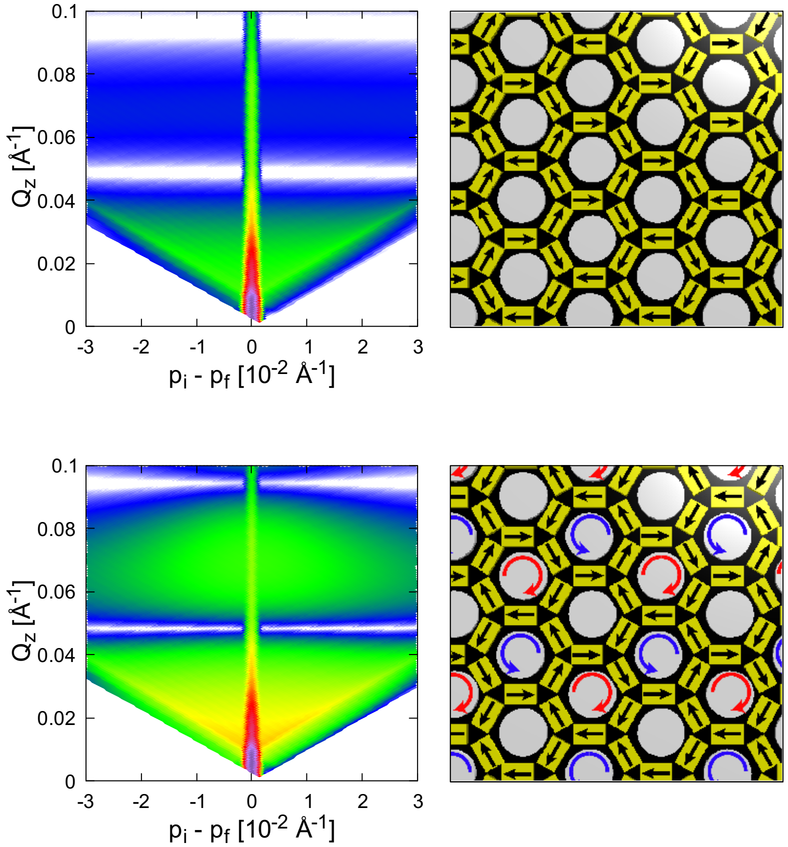

Figure **S7**: Numerically simulated off-specular PNR profiles for the pure states of spin ice and spin solid configurations.

7 Spin configurations with an applied field at low-temperature

The polarized neutron reflectivity measurement at T = 5 K was repeated with an in-plane external magnetic field of H = 0.5 T. The specular reflectivity, spin-asymmetry (SA), and the estimated scattering length density (SLD) of the PNR measurement are shown in Fig. **S8**.

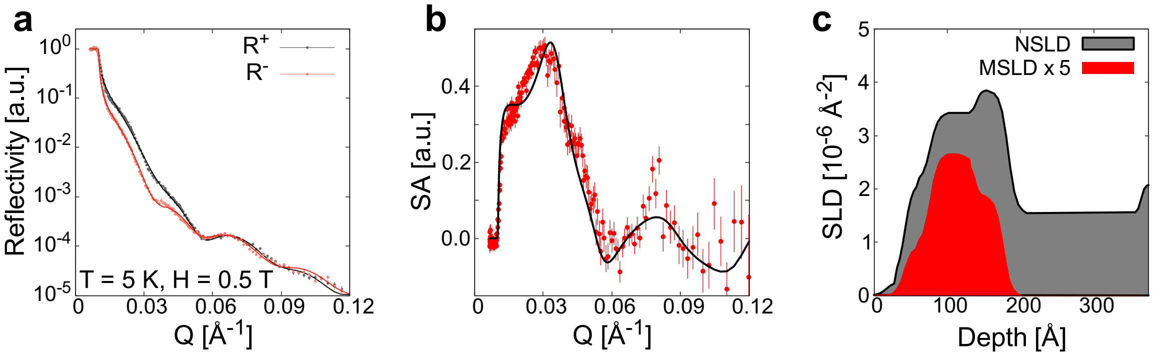

Figure **S8**: **(a)** The measured and fitted specular reflectivity, **(b)** spin-asymmetry (SA) as a function of wave-vector transfer (Q), and **(c)** scattering length density (SLD) of the nuclear (NSLD) and magnetic (MSLD) origins as a function of depths at T = 5 K, and H = 0.5 T

8 Roughness analysis

The roughness analysis from atomic force micrograph is shown in Fig. **S9**. The RMS height difference of the top surface is less than 0.5 nm along any travel direction of connecting honeycomb elements.

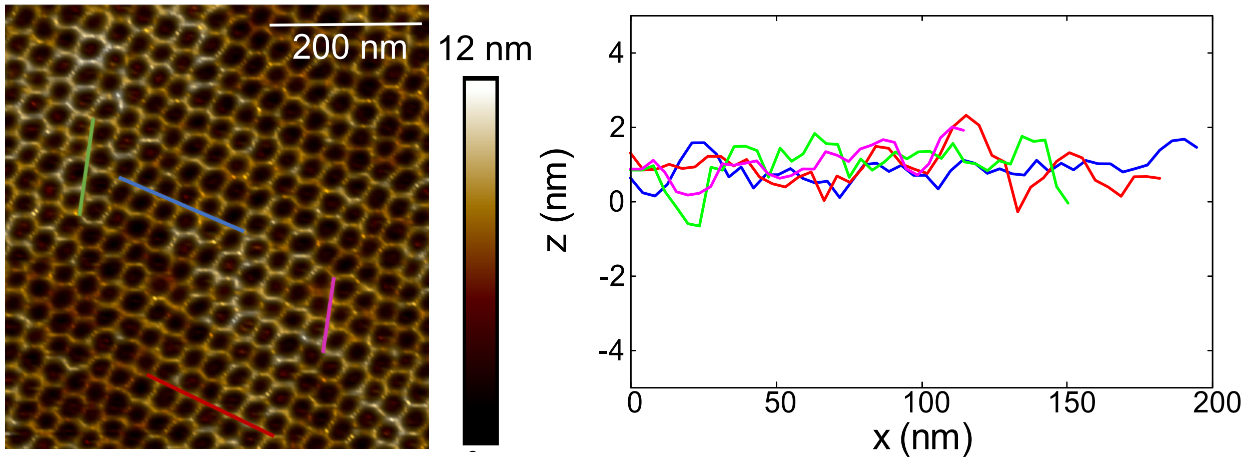

Figure **S9**: Roughness analysis plot of atomic force micrograph.

Correspondence Correspondence and requests for materials should be addressed to D.K.S. (email: singhdk@missouri.edu).
